# The impact of prescriptions audit and feedback for antibiotic use in rural clinics: interrupted time series with segmented regression analysis

**DOI:** 10.1186/s12913-018-3602-z

**Published:** 2018-10-16

**Authors:** Li Zhen, Chenggang Jin, Hao-nan Xu

**Affiliations:** 1 School of Public Affairs, Xiamen University, Room 308 Chengzhi Building, 422 Siming South Road, Siming District, Xiamen, Fujian Province 361005 People’s Republic of China; 2 School of Social Development and Public Policy, Beijing Normal University, 2008 North Main Building, Beijing Normal University, 19 Xinjiekouwai Street, Haidian District, Beijing, 100875 People’s Republic of China

**Keywords:** Segmented regression analysis, Interrupted time series, Antibiotic use, Prescription audit and feedback

## Abstract

**Background:**

Problems of irrational antibiotic use by prescribers are ongoing and have escalated following reductions in the cost of essential drugs policy. In an attempt to improve prescribing practices for village doctors and rational use of essential drugs, a program designed to audit and monitor drug use was established. However, the effects of the program to control antibiotic resistance and changing the village doctors’ prescribing behaviors remain largely unknown. This study measured the effect of the program on levels of antibiotic use.

**Method:**

Data was collected covering a 22-month period, before, during and after the program was implemented in rural clinics. Segmented regression analysis with interrupted time series (ITS) data was used to examine whether there had been a significant interaction with the onset of the program in September 2011 and levels of antibiotic use from November 2010 to August 2012. Both serial and 12-month lag autocorrelations were controlled for.

**Results:**

A noticeable drop about 6.15% per month (95% CI: -13.36%; 1.06%, *P* = 0.089) for the antibiotic use in outpatients, which is lower of effect size assuming that the program has the immediate impact of the program were captured for the immediate effect of the program. Meanwhile, levels of antibiotic use would have continued to decrease by 1.12% per month (*P* = 0.034) as they did in the absence of the program.

**Conclusion:**

The central finding was that the prescription audit and feedback program was associated with significant decreases (*P* = 0.034) in antibiotic use after its implementation.

## Background

The first release of surveillance data on antibiotic resistance of the WHO published in January 2018 highlighted the threat posed by antibiotic resistance to the worldwide. It revealed that around 500,000 people with suspected bacterial infections among 22 countries [1]. As a global serious health threat, the abuse of antibiotics not only accelerates the spread of drug-resistant bacteria, but also undermines people’s resistance to infection, leads to cancer [2]. To prevent antibiotic abuse and reduce the risk of antibiotic resistance, many interventions were undertaken in several countries. With guidance and assistance from the WHO, an increasing number of countries have joined in the national antimicrobial resistance surveillance systems to control antimicrobial resistance. Many governments attempts to develop strategies to contain excessive use of antibiotics, which include educational materials such as standard treatment guidelines or clinical guidelines, flow charts/diagnostic cards and simple forms of printed information [3, 4]. These materials are widely used in settings that involve: face-to-face education, seminar/large group meetings, peer review and feedback, focus group discussion/participatory training and in-service training/supervision [5]. Among these, reviewing patient records with clinical guidelines is commonly used in developed countries. Two studies carried out in Mexico have found that the introduction of peer review and feedback was better in effect than other methods: the prescribers taking part in a peer review committee to discuss prescribing patterns was very successful in both the short and the long term [6]. Moreover, the interactive educational intervention and managerial intervention on improving physician prescribing pattern were affordable and accessible for primary care physicians in Mexico [7]. Previous studies have also shown feedback with discussion had much more significant changes on prescription behaviors than without discussion [8, 9].

Since China started the economic reform in 1978, coverage of health care benefits shank dramatically. Health care facilities stopped receiving state funding since 1980 and began to operate a fee for service model. Physicians had to operate on patient fees, generating income by provision of services and usage of medicines. The more procedures they perform, the more pay checks physicians will get. Fee for service model put both rural and urban residents into vulnerability. It is inevitable that more drugs than needed and higher profit drugs have been prescribed for revenue, and antibiotic sales accounted for a rather large proportion. To address over-prescription of antibiotics, different approaches have been introduced in an attempt over recent years [10], such as implementing antimicrobial stewardship strategies [11], educational interventions [12] and public reporting [13]. However, these strategies did not fully succeed in addressing antimicrobial resistance because there was no influence on the fee for service model. The physicians still rely on over-prescribing and excessive medical examinations to get higher income, and their prescribing behaviors failed improve completely.

**Fig. 1:**
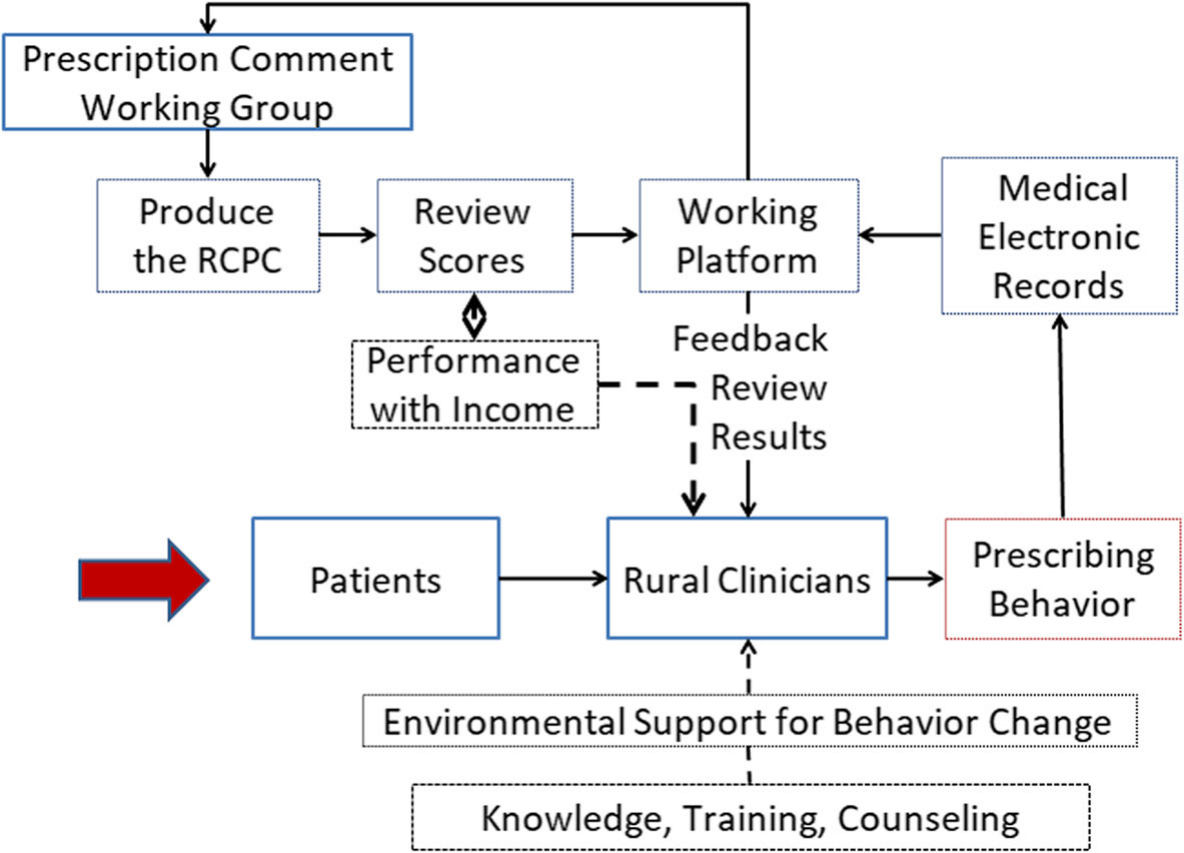
The process of the RCPC plan

In order to prevent physicians applying the fee for service model and improve the quality of prescribing, Huangdao Bureau of Health introduced a prescription audit and feedback plan——the Rural Clinics Prescription Comment (RCPC). This RCPC program separated physicians’ income from prescription drug sales through regulations but with prescribing behaviors. Since September 1st, 2011 the Prescription Comment Working Group (PCWG) has produced the RCPC program every two weeks through the prescription comment working platform. The PCWG is composed of clinical pharmacologists and experienced clinicians, who were selected from town hospitals. The town hospitals are the leading institutions of the rural clinics. The rural clinics are state-owned institutions providing primary care to approximately 800,000 inhabitants of the Huangdao District. The process proceeded as follows and was also presented in Fig. 1. First, the working group sampled the prescriptions of the rural clinics, which constituted at least 20% of the amount of every rural clinic. Second, they marked each selected prescription one by one according to the Guidelines for Peer Review and Feedback of Rural Clinics. The Guidelines were developed by the Prescriptions Comment Expert Group and Leading Group of Huangdao Bureau of Health. The guidelines were about “how to investigate drug use in primary health facilities”, “irrational use of antibiotics”, “promoting rational use of medicines” and “how to comment and mark the prescriptions”. Third, the physicians of every rural clinic would receive the score of the RCPC through the working platform and be made aware of concerns raised by the PCWG. Following this process, the Huangdao Bureau of Health organized workshops and month meetings regularly, giving feedback of the prescription, helping the physicians improve prescribing practices and sharing recommendations and advice intended to solve prescription problems. In addition, face-to-face instruction was offered every month in order to help address severe problems.

The aim of the study is to employ interrupted time series (ITS) methods to investigate the effects on the usage of antibiotics of the introduction of such an approach in Huangdao District of Qingdao in Shandong Province.

## Methods

### The RCPC

#### Design

This is a full-coverage, nonrandomized study of a primary health care intervention designed to analyze the impact of the RCPC program on antibiotic prescribing practices in outpatients. As all the rural clinics received this intervention, there is no control group. So this study applies a pre-experimental study design. In all cases, the outcome was observed every month continuously for an overall period of 22 months. Ten months through November 2010 to August 2011 of observation was undertaken prior to, and twelve months following the introduction of the RCPC through September 2011 to August 2012.

#### Subjects and setting

Although all available records were extracted from the Health Service Information System for the period from September 2011 to August 2012, there were gaps in the records available. The primary reason for these gaps was that many rural clinics were newly built or merged, there were no records collected during the reconstruction period. Accounting for these limitations, this study selected prescription records from a total of 123 rural clinics.

All records were drawn from the patients’ medical electronic documentation, the primary data source. The data was subsequently coded by post-graduate students, and assisted by professors from Beijing Normal University, Treatment coding concentrated on accurately identifying drugs that were antibiotics for both oral and intravenous use intended for systemic use, and comprehensive lists (based on Essential Medicine Lists in each category) were applied to assist the coders.

### Measurements

All the valid prescriptions prescribed during the period were taken into account in final statistical analyses. For the purpose of statistical evaluation, the percentage of encounters with an antibiotic prescribed(using antibiotic use as abbreviation), which was recommended by the WHO [14], was considered for the assessment of the RCPC in this study. It was calculated by dividing the number of patient encounters during which an antibiotic is prescribed, by the total number of encounters surveyed in one month, multiplied by 100.The names and definitions of all variables used in this analysis are listed in Table 1.

**Table 1:** Names and definitions of variables used in the econometric analysis

Variable names	Definitions
Dependent variables	
*y*_*t*_	antibiotic use in month *t*
Independent variables	
*time*	a continuous variable indicating time in month *t* from the start of the study
*intervention*	a dummy variable with a value of 1after the RCPC, 0 otherwise
*postslope*	a continuous variable coded from 1 sequentially after the RCPC, 0 otherwise
*cold*	a dummy variable

### Analysis

The effect of the RCPC program was evaluated by segmented regression analysis. The model this study uses a linear trend in the outcome within each segment. The specification of the linear regression model to be analyzed is as follows:

1 $$ {y}_t={\beta}_0+{\beta}_1\times time+{\beta}_2\times intervention+{\beta}_3\times postslope+{\beta}_4\times cold+{\upvarepsilon}_t $$

In this model, *β*_0_ reflects the baseline level of the outcome, the antibiotic use at the beginning of the period (at time 0); *β*_1_ represents the change in the antibiotic use that is independent from the intervention, which is the structural trend; *β*_2_ captures the change in level of the outcome, representing the antibiotic use after the intervention; and *β*_3_ estimates the change in trend in the antibiotic use after the intervention [15]; *β*_4_ controls for the Spring Festival holiday, which is a circumstantial event. It is when the Spring Festival that is the coldest time of the year in China, and common seasonal illness (cold) prevalence. The possibility of overuse antibiotics rises to treat fever and other associated symptom [16]. The variable *cold* is assigned the value 1 over the period and 0 otherwise. The error term ε_*t*_ at time *t* represents the random variability unexplained by the model. Linear regression model consists of a normally distributed random error and an error term at time *t* that may be correlated with errors at preceding or subsequent time points.

All the tests consider the two-sided significance level of 5%. Stata version 12.0 was used to perform all statistical analyses including autocorrelation. Autocorrelation may lead to underestimated standard errors and overestimated significance of the effects of an intervention and the value of Durbin-Watson statistic close to 2 indicates sign of first-order autocorrelation. In this study, generalized difference method was employed. Assuming there was first-order autocorrelation with *ε*_*t*_, and *ε*_*t*_ = *ρε*_*t* − 1_ + *μ*_*t*_,| *ρ*| < 1, where *μ*_*t*_ was the classical error term.

2 $$ {y}_t={\beta}_0+{\beta}_1{x}_t+{\varepsilon}_t $$

For equation (2), if allowing 1 lag on each endogenous variable, equation (3) would be as follows:

3 $$ {y}_{t-1}={\beta}_0+{\beta}_1{x}_{t-1}+{\varepsilon}_{t-1} $$

If equation (3) was multiplied by *ρ*, then equation (4):

4 $$ {\rho y}_{t-1}=\rho {\beta}_0+\rho {\beta}_1{x}_{t-1}+\rho {\varepsilon}_{t-1} $$

Equation (3) minus equation (4),

5 $$ {y}_{t-1}\left(1-\rho \right)={\beta}_0\left(1-\rho \right)+{\beta}_1\left({x}_{t-1}-\rho {x}_{t-1}\right)+\left({\varepsilon}_{t-1}-\rho {\varepsilon}_{t-1}\right) $$

In equation (5), there is no autocorrelation and *ε*_*t* − 1_ − *ρε*_*t* − 1_ = *μ*_*t*_ is the classical random error term, So the ordinary least squares could be used to get the parameters of best linear unbiased estimation. Suppose ${y}_t^{\ast }={y}_{t-1}-\rho {y}_{t-1}$,${\beta}_0^{\ast }={\beta}_0\left(1-\rho \right)$,${x}_t^{\ast }={x}_{t-1}-\rho {x}_{t-1}$, and then

6 $$ {y}_t^{\ast }={\beta}_0^{\ast }+{\beta}_1\ {x}_t^{\ast }+{\mu}_t $$

Applying generalized difference method to would decrease the sample size from n to n-1. So Prais-Winsten method was employed to make up for this, applying ${\mathrm{Y}}_1^{\ast }=\sqrt{1-{\uprho}^2}{\mathrm{Y}}_1,{\mathrm{X}}_{\mathrm{j}1}^{\ast }=\sqrt{1-{\uprho}^2}{\mathrm{X}}_{\mathrm{j}1}$ (j = 1,2,…,k) to the difference series, which could eliminate the effect of simple size’s change.[6]

## Results

### Descriptive and analytic results

**Fig. 2:**
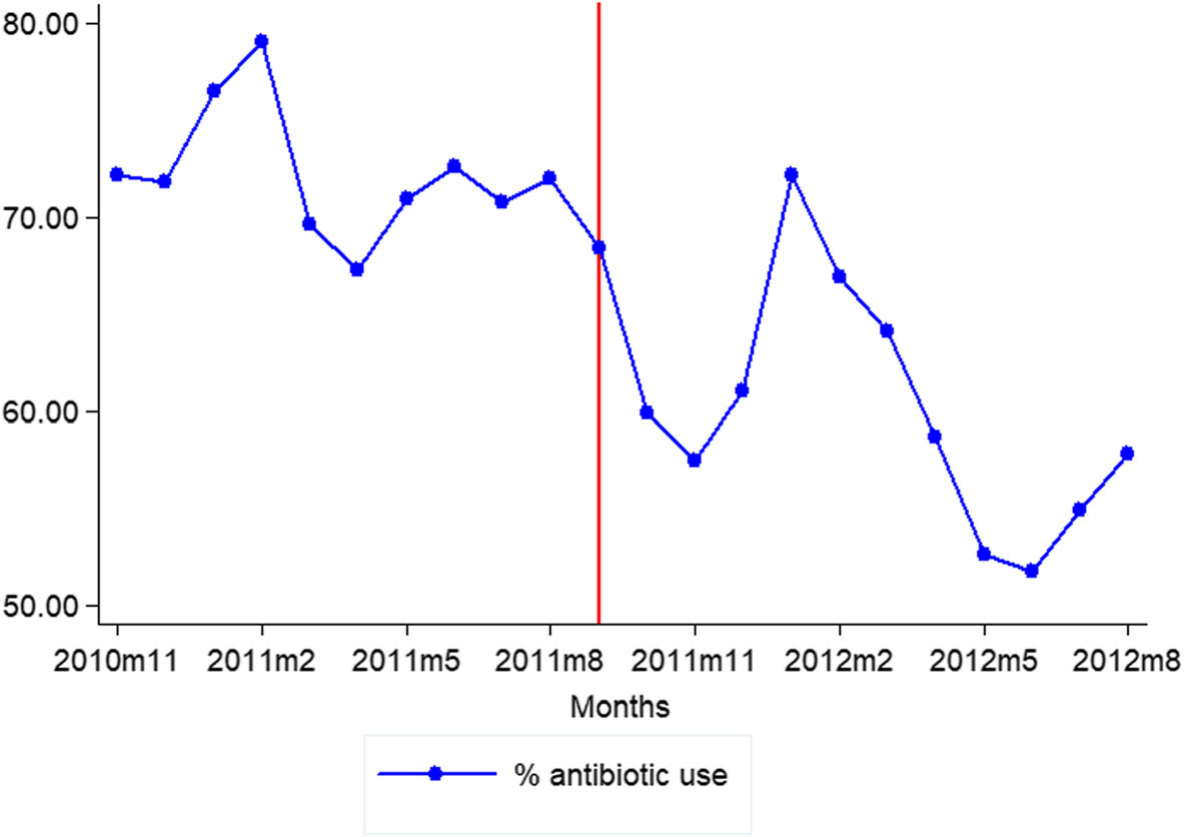
Trend in month-to-month antibiotic use, November 2010–August 2012

The percentage of encounters with a prescribed antibiotic (by month of prescription) that were subject to the intervention is presented in Fig. 2 and Table 2. Both for the antibiotic use, there was a noticeable decrease in the average level of antibiotics utilization prior to and following the intervention. However, there is evidence of seasonality in antibiotics data. During the period of the traditional Chinese calendar, the percentage of encounters with an antibiotic rapidly reached its peak in 2011 and the same months of 2012. Prior to the introduction of the RCPC, there were marked increases in prescriptions towards the beginning of the traditional Chinese calendar. This was followed by a decline in prescribing activity in February of the following year. After introduction of the RCPC, during the peak of the Spring Festival, prescribing activity appeared to have been attenuated.

**Table 2:** the antibiotic use monthly before and after the RCPC program (%)

Months	Before the RCPC program	Months	After the RCPC program
Nov-10	72.22	Sep-11	68.39
Dec-10	71.80	Oct-11	59.88
Jan-11	76.50	Nov-11	57.46
Feb-11	79.03	Dec-11	60.99
Mar-11	69.64	Jan-12	72.16
Apr-11	67.23	Feb-12	66.89
May-11	70.93	Mar-12	64.12
Jun-11	72.61	Apr-12	58.67
Jul-11	70.82	May-12	52.6
Aug-11	71.99	Jun-12	51.67
		Jul-12	54.90
		Aug-12	57.80

### Statistics

For antibiotic use, this regression adjusting for auto-correlation model was highly predictive, yielding a total R^2^ value of 0.83. A Durbin-Watson statistic of 1.923, larger than the upper value of 1.66 and around 2, suggests that results from Model 1 is without autocorrelation (see Table 3). This model indicates that at the beginning of the period of observation, there were on average 68.90% antibiotic use in rural clinics. Prior to the RCPC program, the antibiotic use increased by 0.22% per month, but this month-to-month change before the intervention was not significant (*P* = 0.480). With the intervention, it is estimated that there was no significant immediate change in the level of antibiotic use (*P* = 0.089), such that level of the series dropped by approximately 6.15% for an encounter with an antibiotic prescribed (95% CI; − 13.361, 1.057). While after the RCPC program, this rate had been reduced to 1.12% per month and this change in the underlying trend was statistically significant (*P* = 0.034).

**Table 3:** Effect of the RCPC program on antibiotic use

Independent variables	Coefficient	Std. Err.	t	Significance
*time β*_1_	0.22	0.31	0.72	0.480
*intervention β*_2_	−6.15	3.40	−1.81	0.089
*postslope β*_3_	−1.12	0.48	−2.32	0.034
*cold β*_4_	8.41	1.86	4.53	0.000
*constant β*_0_	68.90	2.46	28.00	0.000

Durbin-Watson statistic (original):1.698

Durbin-Watson statistic (transformed):1.923

## Discussion

This study applies interrupted time series data, using a pre-experimental study design to estimate intervention effects in nonrandomized settings, which is one of the strongest way to capture the effect of the intervention effectively [17]. Although segmented regression analysis can control for prior trends in the outcome and estimate the size of the effect at different time points, as well as changes in the trend of the effect over time, the sources are aggregate prescription data rather than individual-level information. The unit of this study is the monthly antibiotic use, rather than each individual’s percentage of an antibiotic prescribed per month. Using aggregate data may introduce bias due to monthly changes, differences in diagnoses, and inability to analyze individual-level characteristics.

**Fig. 3:**
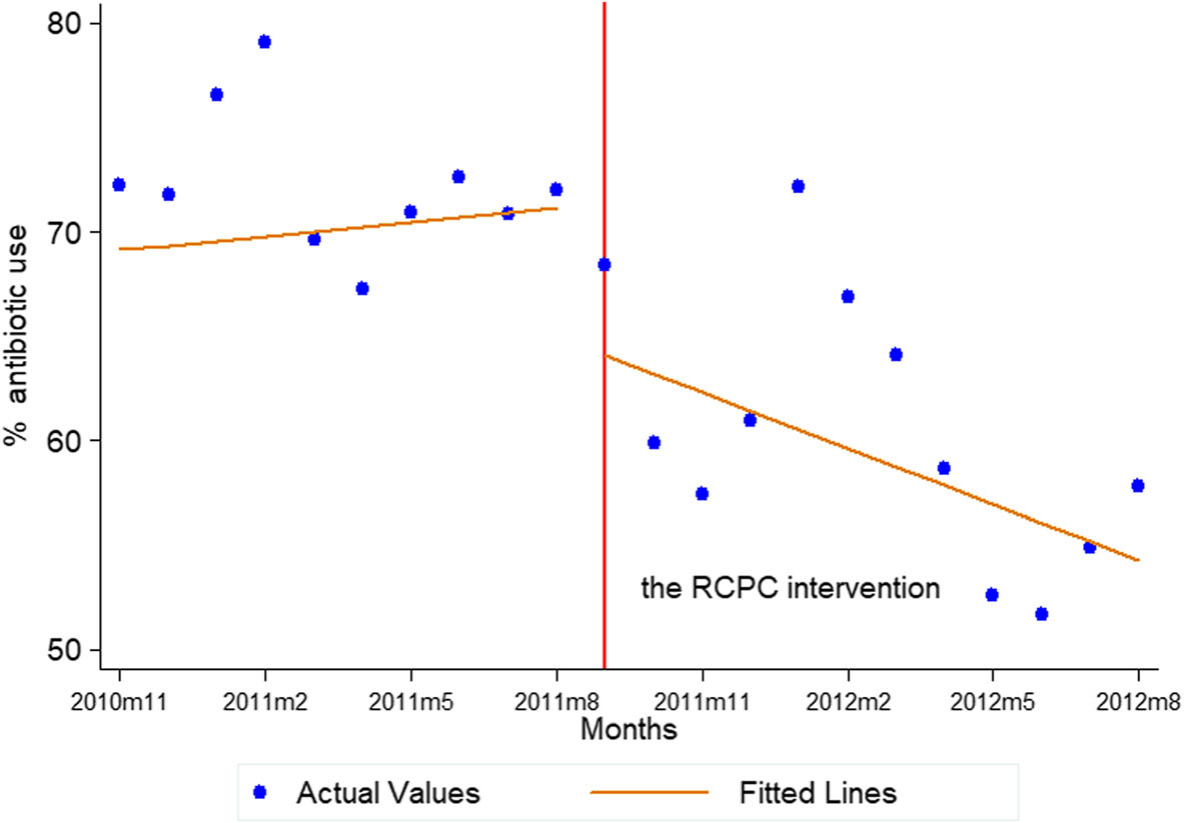
Fitted lines of the segmented regression analysis of interrupted time series for the monthly antibiotic use, November 2010–August 2012

Analysis of the impact of the intervention demonstrated that both immediate and long term changes in the percentage of encounters with a prescribed antibiotic are associated with the RCPC program. As Fig. 3 shows, following the intervention, as the model suggests, even though the antibiotic use is not statistically significant immediately (*P* = 0.089) when the RCPC was introduced, the RCPC program steeply reduced antibiotics prescription rates indeed which dropped significantly and rapidly by about 1.12% per month (*P* = 0.034). These findings strongly suggest that this program can reduce oral antibiotics and combined use of antibiotics and benefit the prescription practices of rural physicians. Examining relative changes in prescription rates prior to and following the intervention highlights the durability of the effect of the RCPC program.

The RCPC program is designed to enhance rational drug prescribing practices by alternating rural physicians’ financial incentives from overprescribing and over-the-counter availability of antibiotics sales to prescription performance. Much of the damage caused by incorrectly prescribed antibiotics, including increased antibiotic resistance, unaffordable economic costs, and lack of compliance were prevented. Furthermore, to improve rural physicians prescribing practices, it strengthened rural clinic supervision, regulated medical practices, and effectively utilized various types of medical resources to identify potential medication problems and evaluate the appropriateness of clinical drug use (including drug indications, drug selection, usage and dosage, drug interactions, incompatibility, etc.). This peer review and feedback program initiative undertaken in Huangdao District improved physicians’ prescribing practices and promoted rural residents’ medical prescription and medication safety. It could be taken as a kind of comprehensive quality management of the medical services offered by rural clinics, which encourages continuous improvements in the quality of care, changing physicians’ prescribing practices. After released from the fee for service model, there are few over-prescriptions. The affordability and accessibility for rural residents to see doctors and receive medical treatment increases, and make “minor illnesses cured within the village, the general illnesses within the town, and serious illness within the city”.

With respect to study limitations, in contrast to the general recommendation that 12 data points prior to, and 12 data points following the intervention should be used [17]. In this study 10 data points pre and 12 data points post intervention were used. However, this number is not relied on estimates of power and it will be improved in the future when further data is available. The data points are monthly prescription totals, by date of dispensation. Additionally, the underlying trend in the total number of prescriptions dispensed very month over the full year remained unchanged.

In conclusion, this study demonstrated that use of a peer review and feedback policy in primary health care facilities could not only decreases the rate of encounters with prescribed antibiotics, but also improves prescribing behavior of general rural clinic physicians. These results will be substantial contributions to the effectiveness of treatments, safety of therapy and quality of health services.

## Conclusion

This study provides evidence that that the prescription audit and feedback program was associated with significant decreases (*P* = 0.034) in antibiotic use after its implementation in long term. Given the increasing antibiotic resistance worldwide, promoting regular prescription audit and feedback program in medical settings might help decline or prevent overprescribed antibiotic.
